# Production, purification and characterization of polyclonal antibody against the truncated gK of the duck enteritis virus

**DOI:** 10.1186/1743-422X-7-241

**Published:** 2010-09-17

**Authors:** Shunchuan Zhang, Jun Xiang, Anchun Cheng, Mingshu Wang, Xin Li, Lijuan Li, Xiwen Chen, Dekang Zhu, Qihui Luo, Xiaoyue Chen

**Affiliations:** 1 Avian Disease Research Center, College of Veterinary Medicine of Sichuan Agricultural University, 46# Xinkang Road, Ya'an, Sichuan 625014, China; 2 Key Laboratory of Animal Disease and Human Health of Sichuan Province, Ya'an 625014, China; 3 Epizootic Diseases Institute of Sichuan Agricultural University, Ya'an, Sichuan 625014, China

## Abstract

Duck virus enteritis (DVE) is an acute, contagious herpesvirus infection of ducks, geese, and swans, which has produced significant economic losses in domestic and wild waterfowl. With the purpose of decreasing economic losses in the commercial duck industry, studying the unknown glycoprotein K (gK) of DEV may be a new method for preferably preventing and curing this disease. So this is the first time to product and purify the rabbit anti-tgK polyclonal antibody. Through the western blot and ELISA assay, the truncated glycoprotein K (tgK) has good antigenicity, also the antibody possesses high specificity and affinity. Meanwhile the rabbit anti-tgK polyclonal antibody has the potential to produce subunit vaccines and the functions of neutralizing DEV and anti-DEV infection because of its neutralization titer. Indirect immunofluorescent microscopy using the purified rabbit anti-tgK polyclonal antibody as diagnostic antibody was susceptive to detect a small quantity of antigen in tissues or cells. This approach also provides effective experimental technology for epidemiological investigation and retrospective diagnose of the preservative paraffin blocks.

## Findings

Duck virus enteritis (DVE) is an acute, contagious herpesvirus infection of ducks, geese, and swans, characterized by vascular damage, tissue hemorrhages, digestive mucosal eruptions, lesions of lymphoid organs, and degenerative changes in parenchymatous organs [1-5]. The causative agent of DVE is duck enteritis virus(DEV), composing of a linear, double-stranded DNA genome with 64.3% guanine-plus-cytosine content, which is higher than any other reported avian herpesvirus in the Alpha-herpesvirinae subfamily[6]. In duck-producing areas of the world where the diseases has been reported, DEV has produced significant economic losses in domestic and wild waterfowl due to mortality, condemnations, and decreased egg production[7].

With the purpose of decreasing economic losses in the commercial duck industry, studying gK of DEV may be a new method for preferably preventing and curing this disease. Because glycoproteins are the major antigens recognized by the infected host's immune system and play an important role in mediating target cell infection, cellular entry of free viruses, and the maturation or egress of the virus [8,9]. Glycoprotein K is one of the major glycoproteins encoded by the DEV-*gK *gene, which is located in the unique long region of the DEV genome. Additionally, gK is capable of inducing a protective immune response in vivo and is responsible for viral binding to the cellular receptor [10,11].

**Figure 1:**
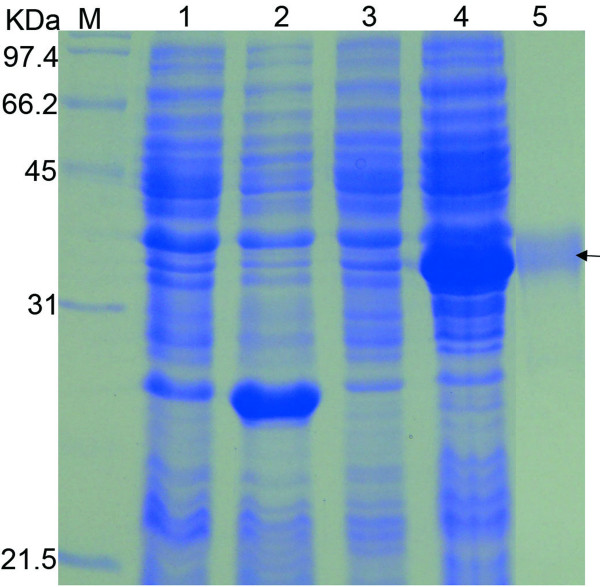
**Expression and purification of the tgK protein**. M represented standard protein molecular weight markers. The arrow marked the purified tgK protein, which was approximately 34.0 KDa according to standard protein molecular weight markers. Lane 1 and Lane 2 respectively represented the uninduced and induced BL21 bacteria within pET-32b(+) plasmid; Lane 3 and Lane 4 respectively represented the uninduced and induced BL21 bacteria within pET-32b(+)/tgK plasmid; Lane 5 was the recombinant tgK protein purified by IMAC.

Although the disease has been reported in 1926, there was little information known about the functions of DEV-gK. To investigate the functions and characteristics of *gK *gene as well as gK, the full-length *gK *gene (*fgK*) and truncated *gK *gene (*tgK*) expression plasmid were constructed[11], only the *tgK *expressed efficiently in prokaryotic system (Figure 1, lane4). The recombinant tgK protein was purified by immobilized metal affinity chromatography (IMAC) and showed in (Figure 1, lane5).

**Figure 2:**
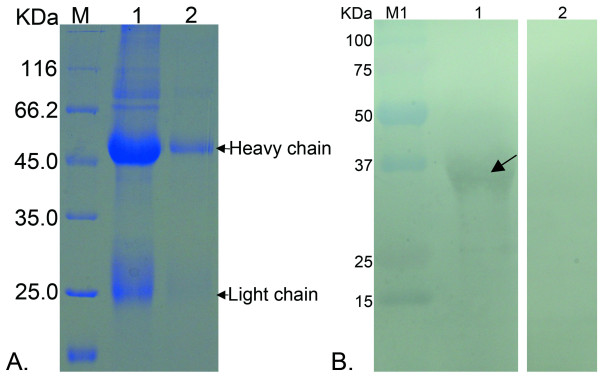
**Purification of the rabbit anti-tgK polyclonal antibody and Western blot assay**. M represented standard protein molecular weight markers; M1 represented bicolor prestained protein markers. **A**. Purification of the rabbit anti-tgK polyclonal antibody. Lane1 represented that the polyclonal antibody was cursorily extracted by saturated ammonium sulfate; Lane 2 stood for the purified polyclonal antibody by ion exchange column chromatography. The heavy chain and light chain were approximately 55 KDa and 22 KDa, respectively. **B**. Western blot assay. Lane 1, Western blotting analysis showed that a specific band was recognized by rabbit anti-tgK monoclonal antibody, which was marked by the arrow; Lane 2, no band was detected by using rabbit preimmune serum.

Then, the purified tgK was used to produce polyclonal antibody. Preimmune serum was collected prior to immunization. New Zealand white rabbits were injected intradermally with a mixture of 0.5 mg purified His-tagged tgK protein mixed with an equal volume of complete Freund's adjuvant (Promega) on the back and proximal limbs (100 μl per site). Two weeks later, the rabbits were boosted twice intramuscularly with 0.75 mg His-tagged tgK protein mixed with an equal volume of incomplete Freund's adjuvant at a one-week interval. Two weeks after the last immunization, the antiserum was harvested from the carotid artery and stored at -70°C for further use[12]. Purification of polyclonal antibody from rabbit serum was initially carried out by precipitation with saturated ammonium sulfate (Figure 2A, lane1). Then, by using the DEAE-Sepharose column (Bio-Rad), the IgG fraction was purified by ion exchange column chromatography following the manufacturer's instructions. The purified IgG fraction was analyzed by 12% SDS-PAGE (Figure 2A, lane2).

Western blotting was used to detect the reactivity and specificity of the tgK. The purified recombinant proteins were separated on 12% SDS-PAGE and transferred onto polyvinylidene fluoride (PVDF) membrane at 120 V for 1.5 h in a BioRad mini Trans-Blot electrophoretic transfer cell (BioRad, Shanghai, China) for western blot analysis. The blotted membrane was blocked at 4°C for 16 h with 10% skimmed milk in TBST (Tris-buffered saline with 0.1% Tween-20, pH 8.0). Then, the membranes were washed and incubated with rabbit anti-tgK polyclonal antibody while using the preimmune serum of normal rabbit as negative control. The membranes were then washed and incubated with horseradish peroxidase-conjugated goat anti-rabbit IgG (Invitrogen) at 1:5000 of dilution in TBST buffer containing 0.5% BSA. After further washing, immunoreactive protein was visualized by using diamino benzidine (DAB). From the result, we can see the purified tgK, which was recognized by rabbit anti-tgK polyclonal antibody, was apparent on western blots (Figure 2B, lane1) as a single specific band approximately 34 kDa. Meanwhile, the rabbit preimmune serum did not show any reaction with tgK in western blots (Figure 2B, lane2). All the data indicated the tgK had good reactivity and specificity.

Enzyme linked immunosorbent assay (ELISA) was used to evaluate the affinity of antibody. Microplates were coated for 1 h at 37°C with 100 μl per well of truncated gK at the concentrations 5 μg/ml in 50 mM carbonate/bicarbonate buffer pH 9.6 and then coated overnight at 4°C. After this procedure, plates were washed three times in PBST (PBS buffer with 0.1% Tween-20) for 5 min each and blocked with 110 μl per well of PBST with 1% BSA for 1 h at 37°C. The sample of the rabbit anti-tgK positive serum was diluted with 11 gradients ranging from 1:800 to 1:819200 and incubated for 1 h at 37°C. After incubating antiserum, plates were washed and incubated with horseradish peroxidase-conjugated goat anti-rabbit IgG (Invitrogen) at working concentration 1:5000 for 1 h at 37°C. After washing 3 times, 100 μl TMB (3,3',5,5'-tetramethyl-benzidine) was added to the plates followed by exposure for 8 minutes. The reaction was terminated with 2 M H_2_SO_4 _and the OD_450 _value was then read with Elx800 Universal Microplate Reader (Bio-Tek Instruments, Inc., Winooski, VT, USA). Also, other plates incubated with rabbit preimmune serum had the same procedures with those plates incubated with rabbit anti-tgK positive serum. The result of ELISA showed a minimum detection limit of the duck anti-tgK positive sera was 1:409600. The higher the titer, the stronger is the affinity. So the affinity of the antiserum collected from rabbits was so good.

The neutralization titer of the rabbit anti-tgK polyclonal antibody was evaluated by micro neutralization test. First of all, duck embryo fibroblasts (DEF) were prepared in 96-well cell culture plate and each well had 250 μl cell suspension. Then, inactivated test sera rabbit anti-tgK (56°C for 30 min) were serially diluted twofold from 1:1 to 1:64. The 200TCID_50 _virus, which was diluted from the virus stock suspension (TCID_50 _= 10^-5.567^), in a 25 μl volume was mixed with an equal volume of serum dilution and incubated at 35°C for 1 h. Also, each serum dilution had 6 duplications. When the cells grew as monolayer, then 50 μl of the incubated mixture was inoculated onto the cells. After a 1 h adsorption period at 37°C, the cells were overlaid with the modified eagle's medium. Meanwhile, seven contrast controls were set up for later observation:1) blank control was normal cells; 2)100TCID_50_, 10TCID_50_, 1TCID_50 _and 0.1TCID_50 _without incubating with diluted positive serum was respectively added to the cells in cell culture plate, used as controls; 3) cells incubated only with high concentration positive serum or negative serum were used as controls. Through observation, the 50% serum neutralized destination was calculated by Reed-muench method[13]. The neutralization titer of the rabbit anti-gK polyclonal antibody was 1:5.623. The result indicated the gK may possess the functions of neutralizing DEV and anti-DEV infection, also has the potential to produce subunit vaccines.

**Figure 3:**
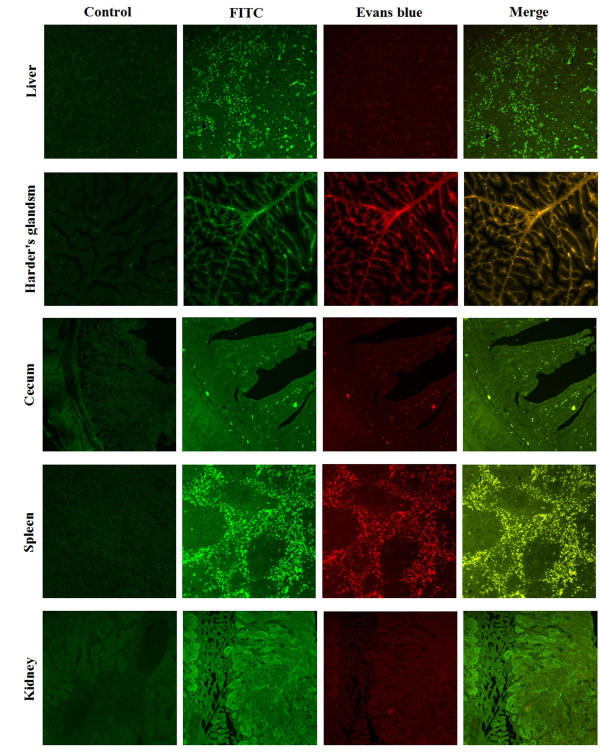
**Indirect immunofluorescent microscopy was used to monitor the DEV antigen distribution in liver, harder's glands, cecum, spleen and kidney of the infected ducks**. The tissue sections were made at 4 μm and stained with an indirect immunofluorescent technique. Images were photographed by using 20× objective. Labels on the left side of this figure indicate different organs from ducks. Negative control is shown in the left of the figure, and the staining methods are indicated above the top horizontal row.

**Figure 4:**
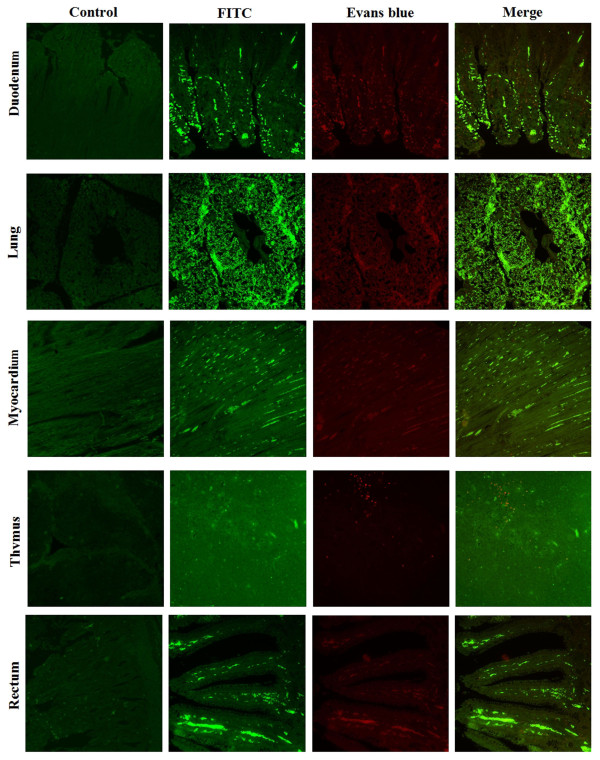
**Indirect immunofluorescent microscopy was used to monitor the DEV antigen distribution in duodenum, lung, myocardium, thymus and rectum of the infected ducks**. The tissue sections were made at 4 μm and stained with an indirect immunofluorescent technique. Images were photographed by using 20× objective. Labels on the left side of this figure indicate different organs from ducks. Negative control is shown in the left of the figure, and the staining methods are indicated above the top horizontal row.

Indirect immunofluorescent microscopy was used to monitor the DEV antigen distribution in the infected ducks by DEV low virulent strain, and thirty-day-old ducks from free pathogen of DEV were used to do this experiment. Some ducks were infected with DEV low virulent strain by intramuscular injection the others were mock-infected with PBS by intramuscular injection as control. After two week post-infection, different tissues were obtained and immediately treated with 4% formaldehyde for 24 h, and then embedded in paraffin. 4 μm thick histological sections were cut from each tissue, mounted, and baked. They were then deparaffinaged and rehydrated in various gradient alcohols. Also, the sections were treated with 0.01 mol/L citrate buffer solution (pH6.0) for 15 min in the microwave oven to restore antigens. Nonspecific binding was prevented by treating the sections with 10% bovine serum albumin (BSA) at 37°C for 20 min. The sections were then treated with 1:100 diluted anti-gK serum for 1 h at 37°C and washed with PBST. Then, they were treated with FITC-conjugated goat anti-rabbit IgG (1:100). Slides were washed three times with PBST, counterstained with Evans blue (0.01% for 3 min), dehydrated, and coverslipped. Images were examined under the Bio-Rad MRC 1024 imaging system[14]. From the result, we can see the DEV antigen in tissues of artificially DEV-infected ducks distributed in the cells of immunological organs and digestive organs such as liver, harder's glands, cecum, spleen, kidney (shown in Figure 3), duodenum, lung, myocardium, thymus and rectum but there was no positive signals in the tissues of mock-infected ducks (Figure 4).

In conclusion, this is the first time to product the rabbit anti-tgK polyclonal antibody and purify the antibody by ion exchange column chromatography. Through the western blot and ELISA assay, the tgK has good antigenicity, and the antibody possesses high specificity and affinity. Meanwhile the rabbit anti-tgK polyclonal antibody has the potential to produce subunit vaccines, and possesses the functions of neutralizing DEV and anti-DEV infection because of its neutralization titer.

Meanwhile, this study showed indirect immunofluorescent microscopy using the purified rabbit anti-tgK polyclonal antibody as diagnostic antibody could be used to detect DEV and antigen location in organs, provide a new diagnostic method to detect DEV, provide useful method and data for researching and clarifying the morbigenous mechanism of DEV.

Until now, there is no report about indirect immunofluorescent microscopy using the purified rabbit anti-tgK polyclonal antibody as diagnostic antibody to detect the antigen locations of DEV in the infected ducks. Indirect immunofluorescent microscopy combines together the special immunoreaction, the good cells morphous maintained in paraffin section with the illuminant easily descried in the black background, which was susceptive to detect a small quantity of antigen in tissues or cells. This approach also provides effective experimental technology for epidemiological investigation and retrospective diagnose of the preservative paraffin blocks.

## Competing interests

The authors declare that they have no competing interests.

## Authors' contributions

SCZ and JX carried out most of the experiments and drafted the manuscript. ACC, MSW, XL, LJL, XWC, DKZ, QHL, XYC helped in experiments and drafted the manuscript. All authors read and approved the final manuscript.
